# Moderate grade subglottic stenosis in children: Laryngotracheal reconstruction versus cricotracheal resection and anastomosis

**DOI:** 10.3389/fped.2022.914892

**Published:** 2022-07-28

**Authors:** Vivianne Beatrix Christina Kokje, Alessandro Ishii, Kishore Sandu

**Affiliations:** ^1^Hôpitaux Universitaires de Genève, Geneva, Switzerland; ^2^Centre Hospitalier Universitaire Vaudois, Lausanne, Switzerland

**Keywords:** subglottic stenosis, laryngotracheal reconstruction, partial cricotracheal resection, pediatric airway, airway surgery

## Abstract

**Objective:**

The surgical treatment of choice of pediatric moderate subglottic stenosis (major grade II and minor grade III SGS or 60–80% lumen obstruction) remains controversial. Laryngotracheal reconstruction (LTR) (with anterior ± posterior grafts for airway expansion) and partial crico-tracheal resection (PCTR) are the mainly described open surgical techniques. We reviewed our pediatric cases with moderate subglottic stenosis to determine the efficacy of LTR versus PCTR.

**Methods:**

A retrospective study of all children between 0 and 18 years that underwent open reconstructive airway surgery between 2012 and 2019. Children who had either acquired or congenital moderate subglottic stenosis (late grade II and early grade III: 60–80% lumen obstruction) were selected.

**Results:**

Twenty-six children with moderate-grade subglottic stenosis were included. Seventeen were treated with LTR and nine with PCTR. No significant differences were observed between LTR and PCTR-treated cases. Decannulation rates were similar, as well as the functional results.

**Conclusion:**

Both LTR and PCTR are valid treatment options for moderate subglottic stenosis. This study indicates to perform the surgery that is most suitable for the characteristics of the patients’ stenosis, the surgeons’ expertise and preference, and the working infrastructure.

## Introduction

Pediatric subglottic stenosis (SGS) is an acquired or congenital disease characterized by a narrowing of the endo-luminal space between the lower end of the vocal cords until the lower border of the cricoid cartilage. The clinical symptoms vary from recurrent croup to stridor, to partial or total airway obstruction requiring a tracheotomy.

Management strategies are largely determined by the severity of the airway obstruction, or the Cotton-Meyer scale: with grade I meaning 0–50% obstruction, grade II: 51–70% obstruction, grade III: 71–99%, and grade IV with no detectable lumen. Furthermore, the European Laryngological Society (ELS) created a classification adding to the Cotton-Meyer scale wherein the authors conclude that the patient comorbidities and the laryngeal sub-site(s) involvement have major implications on the overall surgical success and the patients’ quality of life (1).

Definitive surgical treatment of SGS is aimed at *expanding* the diseased airway or *excising* the diseased part followed by anastomosis of the physiologically normal adjacent airways. The two main open surgical techniques described are laryngotracheal reconstruction (LTR) (with anterior ± posterior rib cartilage grafts for airway expansion) and partial crico-tracheal resection (PCTR) (2).

Patients with grade I SGS are less symptomatic and may only require endoscopic management or, in some cases, only observation. Dense and thick SGS with grades II, III, and IV require open surgery. It is well-established that LTR is preferred in the treatment of *minor grades* of SGS-grades I and II, and some select grade III (3, 4). A PCTR is preferred in *advanced grades* of SGS (III and IV) to eliminate the disease with total resection of the subglottic space with or without the upper trachea (5, 6). A well-mucosalized airway is critical for optimal mucociliary clearance of the airway and is best achieved after PCTR (7, 8). PCTR is a more complex procedure, while LTR is considered more straightforward with minimal morbidity.

Either LTR or PCTR can be suitable for patients with *major* grade II and *minor* grade III SGS, having 60–80% airway obstruction (5–7). This study aims to compare the results of the two surgical techniques for these grades of airway stenosis.

## Materials and methods

### Study design

This retrospective observational study was approved by the institutional review board of the Lausanne University Hospital (CHUV), Switzerland. A database of all patients who have undergone open reconstructive airway surgery has been maintained prospectively since 2005. Children (0–18 years) who had either acquired or had congenital moderate-grade subglottic stenosis (major grade II and minor grade III having 60–80% airway obstruction) were included in the study. Patients with severe comorbidities (cardiac, respiratory, digestive, and neurological deficits) and vocal cord(s) immobility were excluded. All patients underwent either a single- or double-stage laryngotracheal reconstruction or partial crico-tracheal resection. The surgery was performed in a single center, by a single surgeon (KS).

The preoperative evaluation was done as per the European Laryngology Society recommendations (1). Initial examination of the patient was done using flexible bronchoscopy with the child in spontaneous breathing and later by using rigid optics. We observed upper airway dynamics and morphology above the vocal cords, laryngeal mobility, stenosis grade, its craniocaudal length, trachea, and bronchi. A tracheobronchial aspirate was obtained for bacteriological culture. Esophagus and stomach were checked for reflux disease and eosinophilic esophagitis. The presence of any synchronous congenital aero-digestive anomaly/anomalis was noted.

Congenital SGS was assigned to a hypoplastic, small, and thick cricoid cartilage malformation and pathological hypertrophic subglottic mucosa with significant reduction of the airway. The diagnosis was made before intubation. Acquierd SGS had scarred mucosa and history of prior intubation(s).

A single-stage (SS) procedure meant removing the existing tracheostomy at the time of the stenosis treatment. In a double-stage (DS) procedure, the stenosis was treated, and a tracheotomy was performed during the first stage of the treatment. Decannulation was done later in the second stage. Patients with comorbidities were treated in two stages.

Laryngotracheal reconstruction was performed as per the steps described by Monnier (6).

Laryngotracheal reconstruction in a patient who is non-tracheostomized began with laryngeal mask ventilation. The laryngotracheal (LT) framework was exposed and a tracheotomy was performed between the 3–5 tracheal rings. A partial laryngofissure (incision passing below the anterior commissure on the thyroid cartilage, anterior cricoid cartilage, and the first 2 tracheal rings) was made. A midline posterior cricoid split was performed under direct vision taking care of the retro-cricoid mucosa, and the posterior cricoarytenoid muscles. Rib cartilage grafts were harvested from the 7–10 ribs. These grafts were sculpted and fixed with the posterior and anterior cricoid cartilages using 4 or 5 Vicryl with the perichondrium facing the airway. The anterior graft was covered with the thyroid gland to provide support and vascularity. In a SS LTR, a soft Portex blueline endotracheal tube (ETT) was used to support the reconstruction, and the distal tracheotomy was closed, before passing the ETT into the distal airway.

Laryngotracheal reconstruction in a tracheostomized child began passing a flexo-metallic ETT through the tracheostoma. The anterior and posterior cricoid expansion was performed as mentioned above. A Monniers’ LT mold stent was used to support the reconstruction and was fixed intra-laryngeally using 3.0 Prolene. If the tracheostomy was placed high up between the cricoid and up to the 3rd ring, it was shifted more distally leaving at least two tracheal rings from the lower margin of the anterior graft. The tracheostoma was matured using 4.0 or 5.0 Vicryl.

Partial crico-tracheal resection was performed as per our previous publication (8).

Patients, following a SS intervention, were transferred to the PICU, sedated, respired spontaneously, and extubated after 5–7 days. If the airway was judged insufficient for extubation, then the tracheal intubation was prolonged for 2–4 additional days, and then, extubation or tracheotomy was performed according to the airway status. Continuous positive airway pressure through face mask ventilation was often used for the following hours or days in most patients.

After extubation following an SS-PCTR, and depending on the patient’s age and cooperation, sedative agents were used to avoid neck hyperextension for 1–2 additional weeks.

In the case of DS-PCTR, weaning the patients from a ventilator was attempted as soon as possible, but they were kept lightly sedated for additional 7–10 days to keep the neck in flexion.

Endoscopy was routinely planned to verify the airway just before discharge from the hospital.

The Monniers’ LT mold prosthesis was removed under suspension micro-laryngoscopy after 8–12 weeks.

Following decannulation, endoscopies were performed after 3 and 12 months at our institution. The patients were then seen in their own countries and an annual endoscopy was planned with the referring doctor. The most recent results of the endoscopy, respiration, voice, and swallowing were communicated to us by electronic mail.

The patients’ characteristics, operation notes, and postoperative results were obtained by reviewing medical records. The records contained overall decannulation rate (ODR), complications, and pre-and post-operative breathing, including the airway viewed at endoscopy, voice, and swallowing. ODR included surgery-specific success rate plus additional interventions until removal of the tracheostomy cannula. Successful decannulation meant that the patient remained cannula free for a minimum of 2 years. The complications were rated minor (e.g., minor granulations which responded to less than 2 endoscopic treatments) and major (e.g., needed for re-intubation, re-intervention, severe granulations requiring multiple endoscopic procedures ± temporary reinsertion of a laryngeal stent, pneumonia, and re-hospitalization due to respiratory distress). The preoperative data was collected during the hospital stay and the postoperative data recorded were until the last checkup.

The voice was evaluated as per the parent’s response to questionnaires used in a previous publication of our unit (9) and was classified as follows: (1) Normal voice, (2) Mild dysphonia (described as hoarse voice with some difficulties to hear or understand in a noisy environment), (3) Moderate dysphonia (weak voice or ventricular band phonation with easy fatigability), and (4) Severe dysphonia (breathy voice with difficulty to communicate).

Pediatric swallow therapists clinically evaluated the swallowing.

### Statistics

All values are shown as mean (SD) and probability values of *P* < 0.05 were considered statistically significant. The difference between LTR and PCTR was computed for all variables. The database was split and statistical comparison between nominal variables was achieved with Chi2 tests or the Mann–Whitney U-test for the continuous variables. For all tests, 2-tailed *p*-values of <0.05 were considered statistically significant. All statistical analyses were performed with the statistical program SPSS (version 25).

## Results

Twenty-six children with moderate grade subglottic stenosis (Cotton-Meyer stage: major II and minor III: 60–80%) were included in this study (Table 1). The children included presented no concomitant airway anomalies or neurological diseases. The LTR group had more females compared to the PCTR group (*p* = 0.039). The mean age in the LTR group was 1.88 years with an SD of 1.58 versus 1.89 years with an SD of 2.39 in the PCTR group (non-significant). The maximum age in the LTR group was 6 years at the time of surgery and 8 years in the PCTR group.

**TABLE 1:** Patient characteristics.

	LTR	PCTR	*P*-value
Number (% of total)	17 (65%)	9 (35%)	
Age (years)	1.88	1.89	0.634
Male	6 (35%)	7 (78%)	0.579
Congenital	9 (53%)	1 (11%)	*0.037*
Single stage	6 (35%)	3 (33%)	0.920

Immediate post-operative decannulation, by single-stage procedure, was achieved in 35% of the LTR cases and 33% of the PCTR cases. The overall decannulation rate was 94% with LTR and 78% with PCTR. There was no statistical difference in the decannulation rate between the two groups (*p* > 0.05). In the LTR group, 8 children needed additional surgeries (range 1–2 with a mean of 0.63) until decannulation versus 3 children in the PCTR group (range 1–8 with a mean of 1.86) (*p* > 0.05) (Table 1).

Preoperatively, most children had a tracheotomy in place (17 out of 26 patients, 65%), while the others presented with inspiratory stridor (9/26, 35%). Nine single-stage procedures were performed, of which 6 (35%) were in the LTR group and 3 (33%) were in the PCTR group. Seventeen out of 26 patients had a postoperative tracheotomy. At the end of the follow-up period (at 1-year post-surgery), this number dropped to 3 (11%) out of 26 patients; one of the patients with LTR and two of the patients with PCTR are yet to be decannulated (Table 2). One child in the PCTR group was recently diagnosed to have congenital myasthenia gravis and, hence, we opted to keep the tracheostomy at the request of the treating neurologist. He has an age-appropriate permeable airway on endoscopy. The other child has an age-appropriate airway and tolerates a fully capped tracheostomy cannula. However, he could not travel to our center for decannulation due to the COVID-19 pandemic. At the last clinical check-up, stridor or dyspnea was not observed in any of the patients who were decannulated.

**TABLE 2:** Decannulation and surgical complications.

	LTR *n* = 17	PCTR *n* = 9	*P*-value
Direct decannulation (single stage procedure)	6 (35%)	3 (33%)	0.920
Total decannulation	16 (94%)	7 (78%)	0.215
Time to decannulation (days) (median; min-max)	134 (0–825)	434 (0–1497)	0.579
Extra surgeries until decannulation	3 (18%)	3 (33%)	0.174
Complications	10 (59%)	5 (56%)	0.873
Complications major	7 (41%)	2 (22%)	0.075

There was no statistically significant difference between the minor or major complications between the two surgeries (*p* = 1 and *p* > 0.05) (Table 2). Amongst our patients, the mortality rate was zero.

**FIGURE 1:**
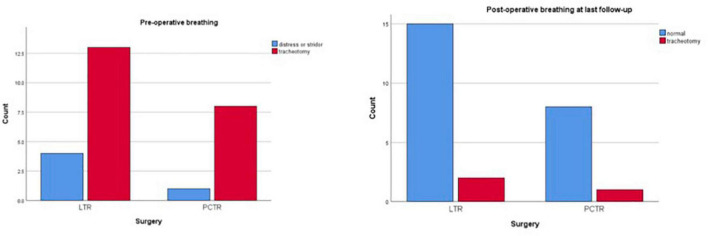
Functional outcomes: breathing.

**FIGURE 2:**
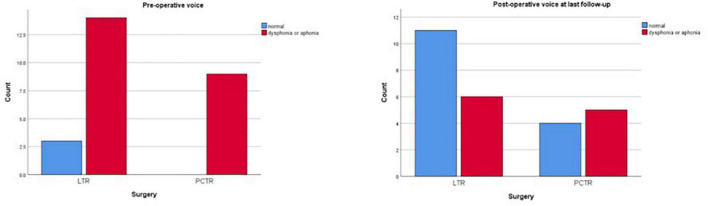
Functional outcomes: voice.

**FIGURE 3:**
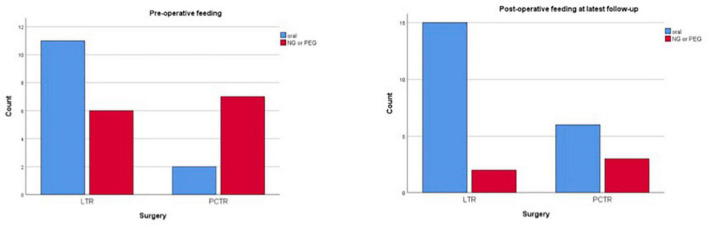
Functional outcomes: feeding.

Both groups reveal significant improvement in pre- and postoperative functions of breathing, swallowing, and voice (Figures 1–3).

Preoperatively, 89% of all patients demonstrated moderate to severe dysphonia or even aphonia (82% LTR, 100% PCTR). Postoperatively, this percentage dropped to a significant overall 39% at the last clinical check-up. Post operatively no significant difference was seen between the LTR and the PCTR group (LTR 32%, PCTR 57%, *p* > 0.05) (Figure 2).

Six out of seventeen (35%) patients with LTR and seven out of nine (78%) PCTR were fed preoperatively with a nasogastric tube or had a percutaneous endoscopic gastrostomy (PEG) in place. During a final clinical check-up, this number dropped drastically to two out of seventeen (12%, LTR) vs. three out of nine (33%, PCTR) (*p* > 0.05) (Figure 3).

### Subgroup analysis

Eight patients with acquired stenosis who were treated with either LTR or PCTR were selected for subgroup analysis. No difference in patient characteristics was found between the groups (Table 3). Preoperatively, 75% of LTR and 100% of patients with PCTR demonstrated moderate to severe dysphonia or even aphonia. Postoperatively, this percentage dropped to 25% in the LTR group and 75% in the PCTR group.

**TABLE 3:** Acquired SGS.

	LTR	PCTR	*P*-value
Age (years)	2.25	2.00	0.574
Male	3 (38%)	6 (75%)	0.131
Direct decannulation (single stage procedure)	3 (38%)	3 (38%)	1.000
Total decannulation	7 (88%)	6 (75%)	0.522
Extra surgeries until decannulation	3 (38%)	1 (13%)	0.380
Complications	5 (63%)	5 (63%)	1.000
Complications major	5 (100%)	2 (40%)	0.131
Pre-operative normal voice	2 (25%)	0 (0%)	0.131
Normal voice at last control	6 (75%)	2 (25%)	0.067
Pre-operative NGT or PEG feeding	4 (50%)	6 (75%)	0.302
Late post-operative NGT or PEG feeding	2 (25%)	2 (25%)	1.000

Subgroup analysis LTR (*n* = 8) vs. PCTR (*n* = 8).

**FIGURE 4:**
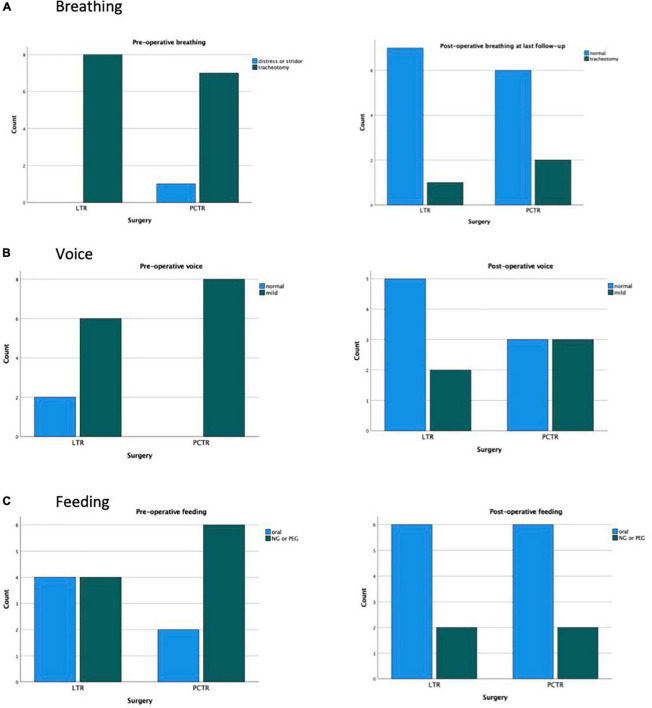
Subgroup analysis acquired SGS. **(A)** Breathing. **(B)** Voice. **(C)** Feeding.

Four out of eight (50%) patients with LTR and six out of eight (75%) patients with PCTR were fed preoperatively with a nasogastric tube or had a percutaneous endoscopic gastrostomy (PEG) in place. At the final clinical check-up, this number dropped to two out of eight (25% LTR) vs. two out of eight (25%, PCTR) (Figure 4).

## Discussion

Endoscopy is the gold standard to diagnose subglottic stenosis (SGS) in children and is treated either endoscopically or by open-surgery operations. Gossamer-thin stenosis, with normal laryngotracheal (LT) cartilage framework and without airway malacia, responds well to endoscopic dilation (using bougies or balloons). Open surgery is required for densely acquired or congenital stenosis, abnormal LT cartilage framework, and associated airway malacia.

Historically, LTR was introduced in the 1970s and has become the treatment of choice for pediatric subglottic stenosis (SGS) with excellent overall results and high decannulation rates in patients with mild-to-moderate isolated uni-level SGS. The overall decannulation rate with LTR is about 85% in grades II and III SGS (4, 10, 11).

The PCTR was introduced in the 1990s and is generally used for severe SGS (late III or IV; >80% airway obstruction) with an overall decannulation rate of more than 90% (5–7, 12).

Monnier (6) suggests that both LTR and PCTR can be options to achieve decannulation in patients with moderate grade SGS. This study complies his findings in patients with major type II and minor type III subglottic stenosis (between 60 and 80% airway obstruction). In terms of functional outcomes, overall decannulation, and complication rates, the surgical treatment by LTR and PCTR are equivalent. The subgroup analysis focused on purely acquired disease and underlines this finding.

This report indicates that the choice of surgery to treat SGS in children should be based on the grade and characteristics of the stenosis, the surgeons’ expertise, preference, and the working infrastructure. The maximal presence of normal subglottic mucosa establishes optimal mucociliary clearance in the immediate and long-term postoperative period and is crucial in the overall surgical outcome. To precisely characterize the SGS, preoperative endoscopy is imperative when choosing the type of surgery. LTR expands the airway using free cartilage grafts and healing is by secondary intention. Such a subglottic wound may be reactivated by an airway infection and thus re-evoke stenosis.

Another important factor to consider when deciding the type of reconstructive surgery is the surgeons’ preference. PCTR is a complex surgery and its success is highly dependent on the surgeons’ training and the working infrastructure. Operative complications like an anastomotic dehiscence and recurrent laryngeal nerve(s) paralysis will lead to severe functional handicaps in the patient. Postoperative management is stressful and, therefore, PCTR must be limited to high-volume centers having adequate experience in these types of surgeries. In comparison to PCTR, the LTR is not as surgeon-dependent, technically less complicated, and has a less challenging post-operative course.

This study has several limitations. The first limitation consists of a selection bias and the limited number of patients in the two groups. Eight out of seventeen (47%) in the LTR group and eight out of nine (88%) in the PCTR group had an acquired disease, meaning that the majority of PCTR was indicated for acquired SGS. The two types of surgeries for a similar grade of stenosis were compared in the study, but we preferred PCTR over LTR in patients with diseased subglottic mucosa. This prompted us to do the subgroup analysis for pure acquired SGS. This analysis revealed that functional outcomes in both groups were comparable and significantly improved post-surgery.

Secondly, it is a retrospective study design. Multicentric studies involving more patients should guide us in the future while making the correct surgical choice.

## Conclusion

In conclusion, for major type II and minor type III SGS, both LTR and PCTR obtain excellent results. Decannulation rates can be improved by appropriate selection of the patient and the type of surgery, optimal execution of the intervention, and optimal postoperative management. Choice of the surgery is dependent on the characteristics of the stenosis, the available volume of normal physiological subglottic mucosa, the expertise of the surgeon, the experience of the multidisciplinary team, and the intra-hospital infrastructure.

## Data availability statement

The original contributions presented in this study are included in the article/supplementary material, further inquiries can be directed to the corresponding author.

## Ethics statement

The studies involving human participants were reviewed and approved by the Institutional Review Board of the University Hospital in Lausanne (CHUV). Written informed consent to participate in this study was provided by the participants’ legal guardian/next of kin.

## Author contributions

VB: preparation manuscript, statistics, and writing and finalizing the manuscript. AI: preparation of data. KS: airway unit head, performed all surgeries, and reviewed the manuscript. All authors contributed to the article and approved the submitted version.
